# Local work function on graphene nanoribbons

**DOI:** 10.3762/bjnano.15.91

**Published:** 2024-08-29

**Authors:** Daniel Rothhardt, Amina Kimouche, Tillmann Klamroth, Regina Hoffmann-Vogel

**Affiliations:** 1 Institute of Physics and Astronomy, University of Potsdam, Karl-Liebknecht-Str. 24–25, 14476 Potsdam-Golm, Germany; 2 current address: Swiss Federal Laboratories for Materials Science and Technology, CH-8600 Dübendorf, Switzerland; 3 current address: Department of Physics, University of Basel, CH-4056 Basel, Switzerland; 4 Institute of Chemistry, University of Potsdam, Karl-Liebknecht-Str. 24–25, 14476 Potsdam-Golm, Germany

**Keywords:** graphene nanoribbons, Kelvin probe force microscopy, local contact potential difference

## Abstract

Graphene nanoribbons show exciting electronic properties related to the exotic nature of the charge carriers and to local confinement as well as atomic-scale structural details. The local work function provides evidence for such structural, electronic, and chemical variations at surfaces. Kelvin prove force microscopy can be used to measure the local contact potential difference (LCPD) between a probe tip and a surface, related to the work function. Here we use this technique to map the LCPD of graphene nanoribbons grown on a Au(111) substrate. The LCPD data shows charge transfer between the graphene nanoribbons and the gold substrate. Our results are corroborated with density functional theory calculations, which verify that the maps reflect the doping of the nanoribbons. Our results help to understand the relation between atomic structure and electronic properties both in high-resolution images and in the distance dependence of the LCPD.

## Introduction

Graphene’s electronic properties are determined by its two-dimensionality as well as by its semimetallic gapless conical band structure [1]. Its electronic behavior depends strongly on the location of the Fermi level with respect to the Dirac point, the center of the Dirac cones [2]. The location of the Fermi level is a measure of the work function with respect to a different energy reference, the vacuum energy. This position can be tuned by gating [3] or by doping, for example, n-doping for graphene on SiC [4–5] and p-doping by Bi, Sb, and Au substrates [2]. Confining graphene to nanostructures [6–7], for example, to graphene nanoribbons (GNRs), that is, few nanometers wide stripes of graphene, opens additional possibilities of tuning the electronic properties by creating quantum-confined states [8] and opening a size-dependent energy gap [6,9]. As in graphene, the Fermi level of GNRs is also strongly influenced by charge transfer between the substrate and the GNR [10], again related to differences in the work function. Here, we take the work function as a local property influenced by local charge, that is, by the local electrochemical potential. GNRs show strong electrostatic effects at their edges [11], where electrostatic forces occur that we expect to modulate the electrons’ local electrochemical potential. Additionally, the chemical state of GNR edges allows one to substantially tune the bandgap [12], which is also related to the work function. GNRs can be synthesized with atomic precision in an ultrahigh-vacuum environment using on-surface synthesis [13]. This synthesis is well known on coinage metals, namely, Cu, Ag, and Au, which possess a high electron density.

To study these unique electronic properties, a suitable method to study the charge transfer, that is, the local work function, between a GNR and a metal substrate at the atomic scale is needed. In general, as detailed above, the local work function can provide evidence for structural, electronic, and chemical variations at surfaces, all related to charge differences; for a review, see [14]. Kelvin probe force microscopy (KPFM), a method derived from scanning force microscopy (SFM), allows one to study the local work function difference of a sample with great accuracy and with atomic resolution [15–20]. In KPFM, a voltage is applied to the tip in order to compensate electrostatic forces occurring between tip and sample. Such electrostatic forces arise from the different positions of the Fermi level in tip and sample, which give rise to charge transfer. In KPFM, the forces are measured by SFM during image acquisition [21–22]. In this way, an image of the local contact potential difference between tip and sample is obtained. This has been shown not only for general surfaces, for example, insulating surfaces, but also for molecules and molecular layers [18,23–25].

Here, we study the local work function difference of graphene nanoribbons fabricated by on-surface synthesis on Au(111). The GNRs can be clearly discerned from the substrate through their topography, but also through their contact potential difference. GNRs have a measured contact potential that is about 100 meV smaller than that of a Au. Variations in the measurement reveal local work function differences, which are ascribed to the Fermi level shift resulting from the charge transfer between the GNR and the Au substrate. Our results indicate that GNRs are positively charged compared to Au. This is confirmed by calculations and by distance-dependent measurements.

## Experimental

The experiments were conducted in an Omicron VT-SFM system (base pressure 2 × 10^−10^ mbar). The Au(111) single crystal substrate (Mateck GmbH) was cleaned by repeated Ar ion sputtering–annealing cycles. The cleanliness of the samples was checked by SFM measurements. Then, 10,10′-dibromo-9,9′-bianthryl (DBBA) molecules were deposited by thermal evaporation (Kentax evaporator) onto the hot (*T*_sample_ = 470 K) sample surface for 10 min. The deposition rate was kept constant using a quartz crystal microbalance. Annealing up to 670 K for 10 min after deposition induced cyclodehydrogenation and the formation of GNRs following [13,26]. The sample was introduced into our SFM attached to the same vacuum chamber, which was cooled down to 115 K using liquid nitrogen. Nanosensors Si tips (resonance frequency *f*_0_ = 158 kHz and longitudinal force constant *c*_L_ = 45 N/m) and PtIr-coated tips (*f*_0_ = 292 kHz, *c*_L_ = 41 N/m) were used for imaging in the frequency modulation (FM) mode operated by a Nanonis electronic system. The tips were cleaned by sputtering (Ar pressure 5 × 10^−3^ Pa, energy 1 keV, 15 min) and annealing up to 375 K for 1–5 h (pressure below 1 × 10^−7^ Pa) prior to measurement. KPFM imaging was performed in parallel to topographic imaging using an AC excitation voltage of *V*_AC_ = 600–900 mV with *f*_AC_ = 166–730 Hz measured by a lock-in amplifier. AC and DC biases were applied to the sample. In general, the polarity of the KPFM measurements depends on whether the voltage is applied to the tip or to the surface and on the polarity of the voltage applied. In order to ensure that the values and the polarity are compatible with previous results [20,27], the measured results were cross-checked on well-known surfaces, that is, Si(111) and Pb on Si(111). For the average taken over several measurements of the local potential difference (LCPD) shown below, we have mainly used PtIr tips and only a few Si tips since the results obtained in a previous work did not show any difference between metal-coated and non-coated Si-tips [27]. For this work, we assume that the non-coated Si-tips were covered by Au from the sample surface because of tip–sample interactions as we have observed a tip–sample contact prior to taking the data used here.

All calculations were done using the Vienna Ab initio Simulation Package [28–29] (vasp-5.4.4) with the PBE functional [30] and a projector-augmented plane-wave basis (PAW) [31–32]. Dispersion forces are included through Grimmes D3 method [33] with Becke–Jonson damping [34] (IVDW = 12). Further, we include non-spherical contributions from the gradient corrections inside the PAW spheres (LASPH = .TRUE.). For all slab calculations, the lowest gold layer was fixed using the optimized bulk lattice constant (*a*_Au_ = 0.2897 nm). The initial positions for the geometry optimizations were chosen according to the structure reported in [35]. Section I of Supporting Information File 1 shows further details about the geometry of the calculations.

We calculate the local work function Φ(*r*) from the Hartree potential *V*_eff_(*r*) corrected by the Fermi energy, that is, Φ(*r*) = *V*_eff_(*r*) − *E*_Fermi_ as done in [20]. We use different constant values of *z* for the LCPD maps. *z* is parallel to *c*, *x* is parallel to *a*, that is, the long axis of the GNR, and *y* is parallel to *b*. The LCPD maps are derived from


[1] $$\Phi^{\left(s\right)}\left(x,y\right)=\Phi \left(x,y,z_{\text{surf}}+s\right),$$

where *z*_surf_ is given by the *z* coordinate of the uppermost carbon atom of the GNR. *s* is varied from 0.17 to 1.2 nm. Additional details about the density functional theory (DFT) calculations performed in this work are given in Supporting Information File 1.

## Results and Discussion

**Figure 1:**
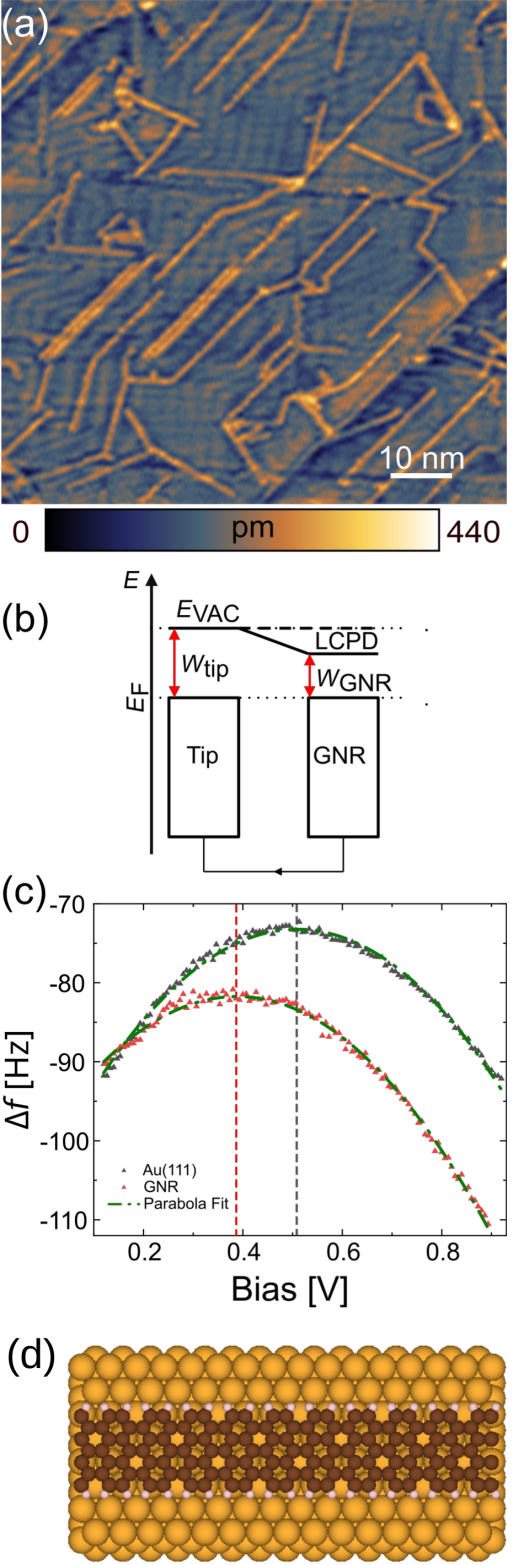
(a) Topography image of GNRs on Au, measured with a Si tip, *f*_0_ = 170.91 kHz, *c*_L_ = 40 N/m, *A* = 1 nm, *Q* = 20,000, and Δ*f* = −21 Hz. (b) In KPFM, local variations in contact potential (CPD) can be measured by applying a voltage between the sample and the AFM tip so that the electric field caused by the CPD is compensated. (c) Δ*f*(*V*) measurements using a PtIr-coated tip along with their second-order polynomial fit measured on GNR and Au. The dashed vertical lines indicate the respective values of the CPD. (d) Scheme of the GNR on Au.

A topographic image of GNRs on the Au surface is shown in Figure 1a. While most GNRs are attached to gold step edges or to other ribbons, we additionally observe isolated individual ribbons. When the tip and the GNR are brought close together, electrostatic forces between tip and sample can be measured. Also, charges can equilibrate, the Fermi levels of tip and surface align, accompanied by an electron flow to the Au, and the GNR is charged, leading to additional electrostatic forces (Figure 1b). During imaging, a voltage is applied in order to compensate for these additional electrostatic forces at each point of the image, leading to a LCPD map.

In Figure 1c, Δ*f*(*V*) curves measured above a GNR and Au are shown. The maxima of the parabolae fitted to the measured data yield the difference of the LCPD values, Δ*V* = 120 mV. Since no impurities have been introduced, the LCPD indicates a charge transfer from the substrate (“p-type doping”), also seen in bulk graphene on a gold substrate [2,36–38].

**Figure 2:**
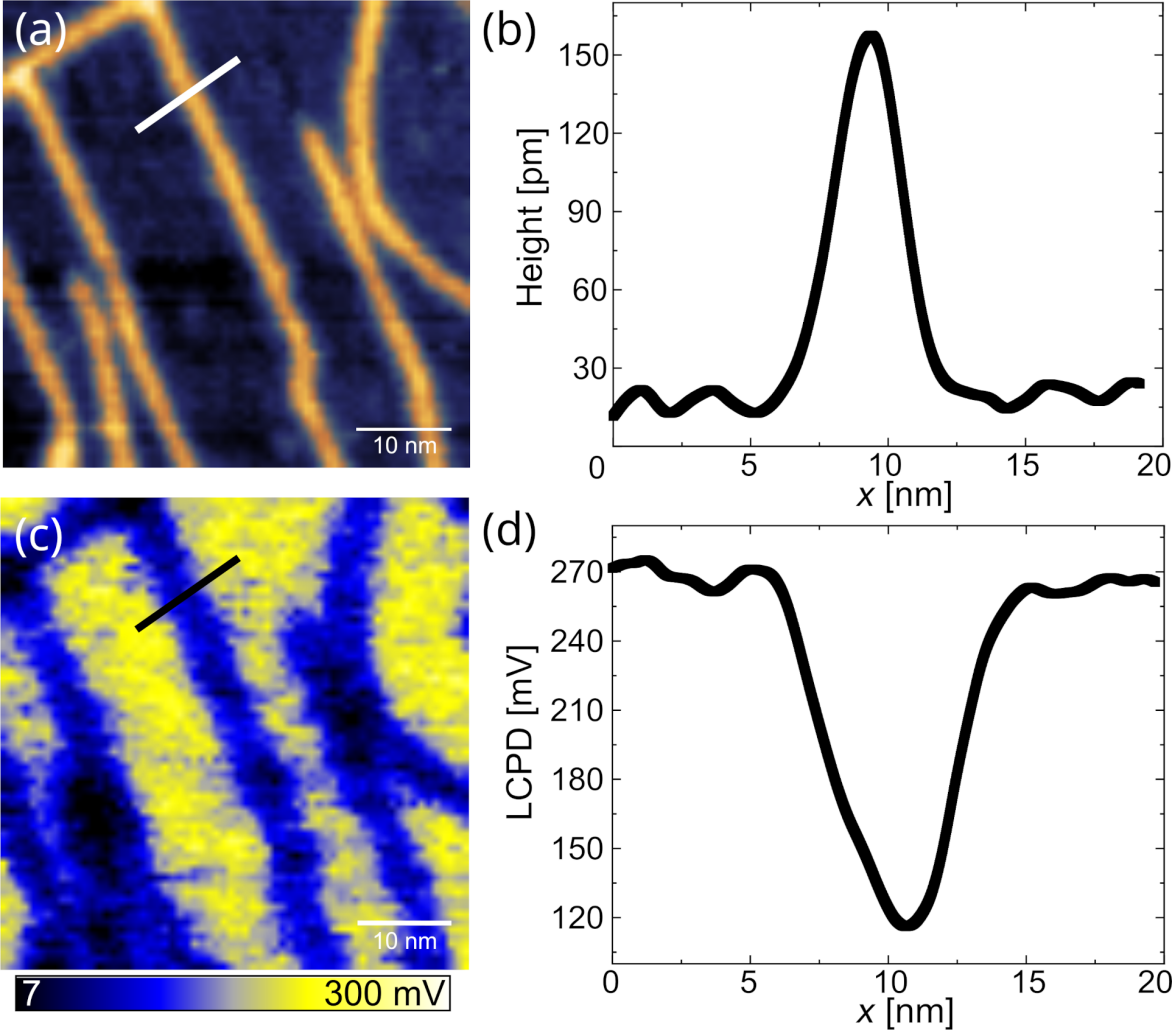
(a) Topography of GNR’s and the Au(111) herringbone reconstruction, PtIr-coated tip, *f*_0_ = 291.52 kHz, *c*_L_ = 41 N/m, *A* = 3 nm, *Q* = 21 000, and Δ*f* = −45 Hz. (b) Line cut through the topographic image at the position indicated by the white line. (c) LCPD image recorded simultaneously with the topographic image, *f*_AC_ = 730 Hz and *V*_AC_ = 900 mV. (d) LCPD line profile taken across a ribbon (black).

Figure 2 shows a topographic image of GNRs on Au(111) surface and its associated LCPD map. In the LCPD map (Figure 2c), GNRs appear as blue stripes on the yellow Au(111) background. From the line profile taken across the ribbon indicated in Figure 2d, we deduce a contact potential difference of 145 mV between the GNR and the Au surface. In Figure 2c, some inhomogeneities of the LCPD along the GNR can be observed, with darker regions appearing along its length. Additionally, some irregularities such as kinks or defects at the edge are observed in the topography measurement. For example for the GNR where the cross section has been taken, marked by a black line, there is a kink associated with a darker region in the local work function, and in the topography image there are some small bright extensions at the side of the GNR also associated with darker regions of the LCPD of the GNR. The electronic states of kinks in GNRs have been studied on a narrower type of GNR in [39]. Only small modifications of their electronic structure have been found. Here, we show that small structural modifications and the associated changes of the electronic states additionally cause a change in the local work function.

**Figure 3:**
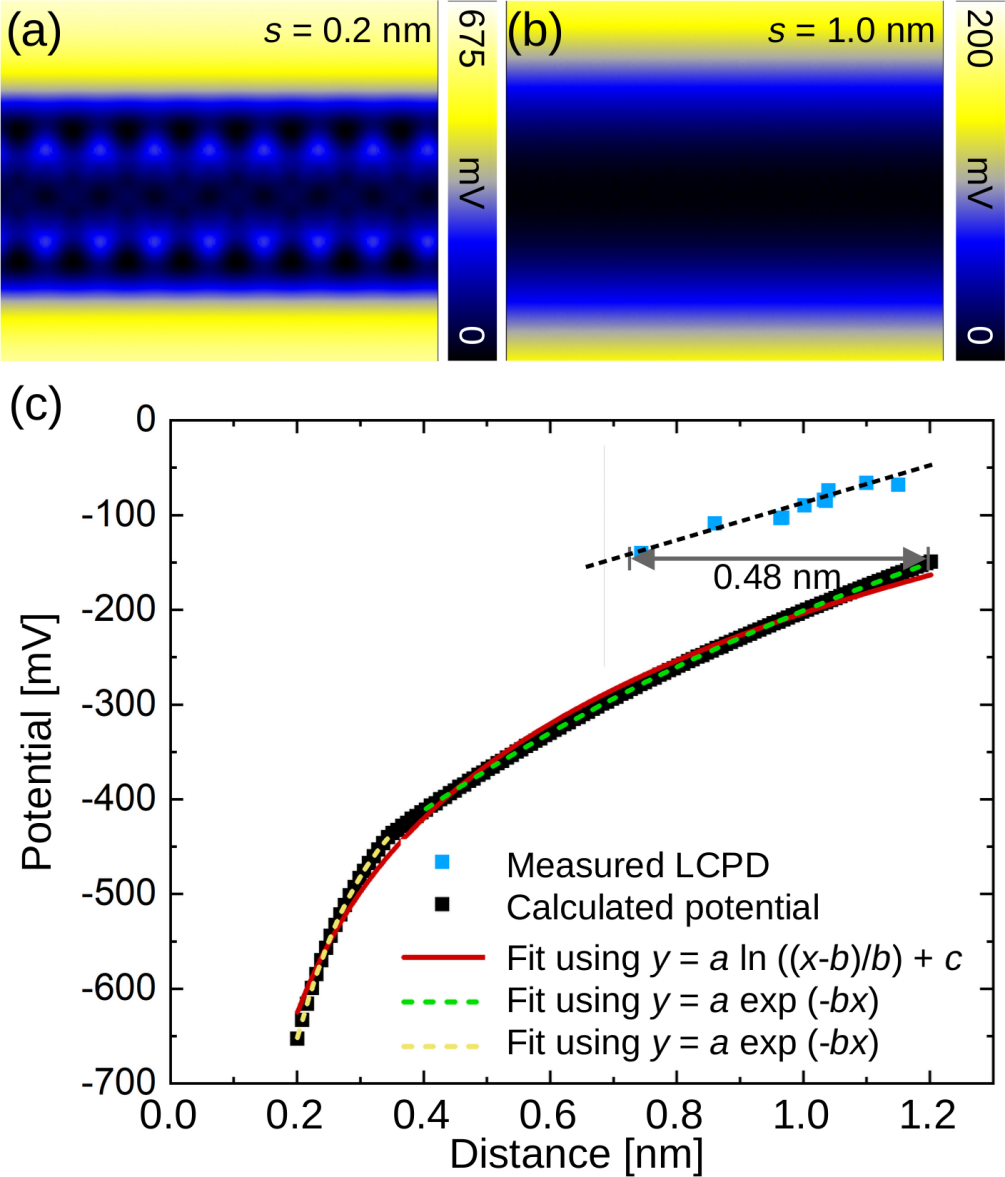
(a,b) Calculated LCPD maps at distances of *s* = 0.2 and 1.0 nm from the GNR plane. (c) 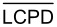 values on GNR/Au (blue squares) versus distance in comparison with calculated local potential values (black squares). The dashed black line serves as a guide to the eye, whereas the red, dashed green, and dashed yellow curves are fits to the data as described in the text.

Figure 3a,b shows local work function difference maps calculated from the Hartree potential of GNR/Au(111). To match the calculated and the measured values, it is necessary to take the difference with respect to a point of reference, here, the Au surface. In the calculations we did not represent the Au herringbone reconstruction, because this is computationally very demanding [40]. At large distances (Figure 3b), the GNR appears as a featureless depression. When the surface is approached and the distance *s* is reduced, the GNR submolecular structure is observed with increasing intensity (see Figure 3a,b and Supporting Information File 1, Figure S1d).

To obtain a more detailed understanding of the charge transfer and for comparison of the experimental results with calculations, we have performed measurements at different frequency shifts. We have then measured a force–distance curve to match each frequency shift to a distance to the sample surface (see Supporting Information File 1, Section II). We show the data in Figure 3c together with the calculated results. With this approach using an average over several measurements (over 257 LCPD line scans), the influence of the tip and sample microstructures on the resulting overall values is minimized. Additionally, 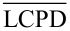 is measured with respect to the reference LCPD recorded on the Au(111) surface to account for variations of the tip contact potential. Again the LCPD of the GNR is negative, and the predicted hole doping is confirmed. In Figure 3c, the 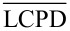 values exhibit a slow decrease towards more negative values with decreasing tip–sample distance. Depending on the tip–sample distance, 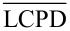 varies from 130 mV for 0.74 nm tip–sample distance to 70 mV for 1.18 nm. These features are consistent with previous results revealing a distance dependence of the LCPD or the electrostatic forces [41–44].

In general, we expect an exponential decay of the electrostatic field 2D Fourier components, where the decay constant λ is the lateral wavelength of the respective 2D Fourier component [45], that is,


[2] $$\Phi \left(z\right)=A_{0}\exp\left(-\left(z-z_{0}\right)/\lambda \right)+\Phi_{0},$$

where *A*_0_ and *z*_0_ are parameters to adjust the tip–sample distance, one of which is redundant, λ is the decay constant, and Φ_0_ is a parameter that allows one to adjust for a different choice of the zero level for voltages. This Fourier analysis could be done for any arrangement of charge and is often practical to find out the main distance dependence. Here, we expect a log-dependence of the electrostatic potential on distance because of the shape of the nanoribbon. Both views are complementary as the logarithm arises from adding over a large number of Fourier components.

Previously, it has been shown that a line charge is a good approximation for electrostatic forces above graphene nanoribbons [41]. For a cylindrical charge with radius *R*, we expect an electrostatic potential that varies as


[3] $$\Phi \left(z\right)=\frac{\rho }{2\pi \varepsilon_{0}}\ln\left(\frac{z-R}{R}\right)+\Phi_{0},$$

where ρ is the charge per unit length [46]. We have fitted this function to the calculated data (black squares in Figure 3c) and obtained ρ/2πε_0_ = 203 mV, *R* = 0.087 nm, and Φ_0_ = −680 mV, represented as a red line. The radius *R* represents the height of the graphene nanoribbon. The charge is ρ = *Q*/*l* = 1.13 × 10^−11^ C/m and corresponds to 0.070 e/nm. The description of the data by the fitted function is good, but a slightly better choice is to describe the DFT-calculated curve by two exponential functions, one with decay length λ = 0.12 nm at close distance, arising from an intramolecular atomic-scale contrast, and a second one with a decay length of λ = 1.24 nm at far distance, resulting from the size of the graphene nanoribbon.

The experimental results (blue squares in Figure 3) generally follow the shape of the calculated curve with a shift. There are several possibilities to understand the origin of this shift. First, we discuss the possibility of a *z* shift between experiment and calculated results. As shown in Figure 3c, a 0.48 nm shift in distance would be needed for experiment and calculations to match. This distance corresponds to the screening length of about one Fermi wavelength (λ*_F_* ≈ 0.52 nm) in Au, calculated from a Fermi energy of 5.53 eV and obtained by the assumption that each Au atom contributes one electron to the Fermi sea. In the calculations, the potential at a certain point in space is calculated, corresponding to a point-charge tip. In the experiment, the tip is either a Si tip or a metal-coated tip. For a perfect metallic layer on the tip, we expect that it adopts an image charge distribution that generates a similar electrostatic field as the charge located in the sample. The charge distribution in the sample in our own calculations is distributed over two atomic layers (Au–Au distance: 0.28 nm, see also Supporting Information File 1, Section III); hence, understanding the 0.48 nm shift as the apparent distance between the charge distribution in the tip and the tip apex is a reasonable assumption. In addition, we compare the shift to the value obtained in [41], 1.7 nm for the total distance, where the tip was composed of graphene nanoribbons with a longer screening length compared to Au. We conclude that the tip used in the experiments shown in this work is “sharp” concerning the electrical measurements in the sense that the charge distribution resides close to the tip apex.

A second way of understanding the shift between experiment and calculations is based on averaging effects [47]. Above, we have used a *z* shift to describe the data, where the difference could be understood as a shift in the potential. The tip exposes its three-dimensional shape to the sample, and the forces result from the electrostatic field of the sample interacting with the tip at each point in space. The averaging effects depend on both the tip sharpness and the tip–surface distance. Here, we expect the relatively large tip radius of metal-coated tips (typically 20 nm) with respect to the width of the ribbons leading to averaging over a considerable part of the Au surface in addition to the GNR and to a reduction of the CPD values due to the long range nature of the electrostatic force.

## Conclusion

In summary, we have imaged graphene nanoribbons using KPFM. We confirm the p-type doping of the GNRs on the Au substrate. The measured LCPD values exhibit a slow decrease with tip–sample distance in qualitative agreement with calculations. Our results highlight the potential of Kelvin probe force microscopy to simultaneously study structural and electronic properties of GNRs and the capability of KPFM as a useful tool for observing the electronic properties in nanoelectronics.

## Supporting Information

File 1 Additional information on the DFT calculations, on the force–distance data used for transforming frequency shift information into distance information, and on calculated charge differences.

## Data Availability

All data that supports the findings of this study is available in the published article and/or the supporting information to this article.

## References

[1] Castro Neto A H, Guinea F, Peres N M R, Novoselov K S, Geim A K (2009). Rev Mod Phys.

[2] Gierz I, Riedl C, Starke U, Ast C R, Kern K (2008). Nano Lett.

[3] Koch M, Ample F, Joachim C, Grill L (2012). Nat Nanotechnol.

[4] Kedzierski J, Hsu P-L, Healey P, Wyatt P W, Keast C L, Sprinkle M, Berger C, de Heer W A (2008). IEEE Trans Electron Devices.

[5] Gu G, Nie S, Feenstra R M, Devaty R P, Choyke W J, Chan W K, Kane M G (2007). Appl Phys Lett.

[6] Li X, Wang X, Zhang L, Lee S, Dai H (2008). Science.

[7] Narita A, Wang X-Y, Feng X, Müllen K (2015). Chem Soc Rev.

[8] Hämäläinen S K, Sun Z, Boneschanscher M P, Uppstu A, Ijäs M, Harju A, Vanmaekelbergh D, Liljeroth P (2011). Phys Rev Lett.

[9] Ritter K A, Lyding J W (2009). Nat Mater.

[10] Vanin M, Mortensen J J, Kelkkanen A K, Garcia-Lastra J M, Thygesen K S, Jacobsen K W (2010). Phys Rev B.

[11] Wang Z, Scharstein R W (2010). Chem Phys Lett.

[12] Wagner P, Ewels C P, Adjizian J-J, Magaud L, Pochet P, Roche S, Lopez-Bezanilla A, Ivanovskaya V V, Yaya A, Rayson M (2013). J Phys Chem C.

[13] Cai J, Ruffieux P, Jaafar R, Bieri M, Braun T, Blankenburg S, Muoth M, Seitsonen A P, Saleh M, Feng X (2010). Nature.

[14] Melitz W, Shen J, Kummel A C, Lee S (2011). Surf Sci Rep.

[15] Nonnenmacher M, O’Boyle M P, Wickramasinghe H K (1991). Appl Phys Lett.

[16] Kikukawa A, Hosaka S, Imura R (1996). Rev Sci Instrum.

[17] Jacobs H O, Leuchtmann P, Homan O J, Stemmer A (1998). J Appl Phys.

[18] Barth C, Foster A S, Henry C R, Shluger A L (2011). Adv Mater (Weinheim, Ger).

[19] Sadewasser S, Jelinek P, Fang C-K, Custance O, Yamada Y, Sugimoto Y, Abe M, Morita S (2009). Phys Rev Lett.

[20] Pérez León C, Drees H, Wippermann S M, Marz M, Hoffmann-Vogel R (2016). J Phys Chem Lett.

[21] Giessibl F J (2003). Rev Mod Phys.

[22] García R, Pérez R (2002). Surf Sci Rep.

[23] Mohn F, Gross L, Moll N, Meyer G (2012). Nat Nanotechnol.

[24] Zerweck U, Loppacher C, Otto T, Grafström S, Eng L M (2005). Phys Rev B.

[25] Neff J L, Milde P, Pérez León C, Kundrat M D, Eng L M, Jacob C R, Hoffmann-Vogel R (2014). ACS Nano.

[26] Schneider S (2016). Herstellung von Graphenstreifen und ihre Untersuchung mit Rasterkraftmikroskopie.

[27] Späth T, Popp M, Pérez León C, Marz M, Hoffmann-Vogel R (2017). Nanoscale.

[28] Kresse G, Furthmüller J (1996). Comput Mater Sci.

[29] Kresse G, Furthmüller J (1996). Phys Rev B.

[30] Perdew J P, Burke K, Ernzerhof M (1996). Phys Rev Lett.

[31] Blöchl P E (1994). Phys Rev B.

[32] Kresse G, Joubert D (1999). Phys Rev B.

[33] Grimme S, Antony J, Ehrlich S, Krieg H (2010). J Chem Phys.

[34] Grimme S, Ehrlich S, Goerigk L (2011). J Comput Chem.

[35] Liang L, Meunier V (2012). Phys Rev B.

[36] Giovannetti G, Khomyakov P A, Brocks G, Karpan V M, van den Brink J, Kelly P J (2008). Phys Rev Lett.

[37] Wofford J M, Starodub E, Walter A L, Nie S, Bostwick A, Bartelt N C, Thürmer K, Rotenberg E, McCarty K F, Dubon O D (2012). New J Phys.

[38] Leicht P, Zielke L, Bouvron S, Moroni R, Voloshina E, Hammerschmidt L, Dedkov Y S, Fonin M (2014). ACS Nano.

[39] Kimouche A, Ervasti M M, Drost R, Halonen S, Harju A, Joensuu P M, Sainio J, Liljeroth P (2015). Nat Commun.

[40] Hanke F, Björk J (2013). Phys Rev B.

[41] Schneider S, Hoffmann-Vogel R (2020). Nanoscale.

[42] Kawai S, Glatzel T, Hug H-J, Meyer E (2010). Nanotechnology.

[43] Krok F, Sajewicz K, Konior J, Goryl M, Piatkowski P, Szymonski M (2008). Phys Rev B.

[44] Enevoldsen G H, Glatzel T, Christensen M C, Lauritsen J V, Besenbacher F (2008). Phys Rev Lett.

[45] Feynman R, Leighton R B, Sands M L (2013). The electrostatic field of a grid. The Feynman Lectures on Physics, Vol. II.

[46] Kuchling H, Kuchling T (2022). Taschenbuch der Physik.

[47] Glatzel T, Sadewasser S, Lux-Steiner M C (2003). Appl Surf Sci.

