# Supplementary material for: Local work function on graphene nanoribbons
Source: Beilstein J Nanotechnol. 2024 Aug 29;15:1125–31. doi: 10.3762/bjnano.15.91 (PMC11368052; doi:10.3762/bjnano.15.91)
Supplement: File 1 — Additional information on the DFT calculations, on the force–distance data used for transforming frequency shift information into distance information, and on calculated charge differences. [file Beilstein_J_Nanotechnol-15-1125-s001.pdf]

BEILSTEIN JOURNAL OF NANOTECHNOLOGY

Supporting Information

for

Local work function on graphene nanoribbons

Daniel Rothhardt, Amina Kimouche, Tillmann Klamroth and Regina Hoffmann-Vogel

Beilstein J. Nanotechnol. **2024,** *15,* 1125–1131. doi:10.3762/bjnano.15.91

Additional information on the DFT calculations, on the force–distance data used for transforming frequency shift information into distance information, and on calculated charge differences

License and Terms: This is a supporting information file under the terms of the Creative Commons Attribution License (https://creativecommons.org/licenses/by/4.0). Please note that the reuse, redistribution and reproduction in particular requires that the author(s) and source are credited and that individual graphics may be subject to special legal provisions.
The license is subject to the *Beilstein Journal of Nanotechnology* terms and conditions: (https://www.beilstein-journals.org/bjnano/terms)

Details on calculations

Convergence tests were made for a small orthorombic unit cell, that is, $a = 3 \times a_{\mathrm{Au}}$, $b = 3\sqrt{3} \times a_{\mathrm{Au}}$, and c set to a value such that about 15 Å of vacuum is between the slabs. Here, c is pointing in the surface normal direction (z), a is the direction of the GNR (x), and b provides about 5.8 Å spacing between two GNRs (smallest distance between two hydrogen cores). We used up to seven gold layers, cutoff energies up to 700 eV, and k-point grids up to $7 \times 7 \times 1$. We found the optimized geometries and the LCPD maps just above the surface, that is, for $s = 1.7$ (van der Waals radius of carbon), to be converged for three gold layers with a cutoff energy of 400 eV and a $4 \times 4 \times 1$ k-point grid.

In order to calculate the LCPD maps further away from the surface, that is, up to 12 Å as given in Figure 3 of the main manuscript, a much larger unit cell is needed. Therefore, we increased of the vacuum gap between the slabs to about 40 Å. Test calculations showed that the LCPD values further away from the surface are rather sensitive to dipole and quadrupole corrections needed for a non-symmetric cell. Further, the LCPD values depend on the spacing between the GNRs in the periodic calculations because for large values of z, the Hartree potential is influenced by the GNRs in neighboring cells. Therefore, we used an inversion symmetric cell with five gold layers in total and a spacing between the GNRs of about 40 Å. Test calculations with only partly optimized geometries indicated that for this spacing, the LCPD values up to $s = 12$ Å are fairly converged.

The unit cell used in the final calculations with a k-point grid of $4 \times 2 \times 1$ and a cutoff energy of 400 eV is shown in Figure S1 ($a = 3 \times a_{\mathrm{Au}}$, $b = 10\sqrt{3} \times a_{\mathrm{Au}}$ and $c = 54.2$ Å). Figure S1a shows the large orthorombic unit cell where all atomic positions are optimized, expect the ones of the lowest gold layer. Figure S1b shows a 4×1 surface unit cell, which corresponds to the LCPD maps shown in Figure S1d and Figure 3 of the main manuscript. Also indicated in Figure S1b are the horizontal planes used to determine the LCPD maps for $s = 2$ Å (blue) and $s = 10$ Å (yellow). A top view of the same cell as in Figure S1b is shown in Figure S1c.

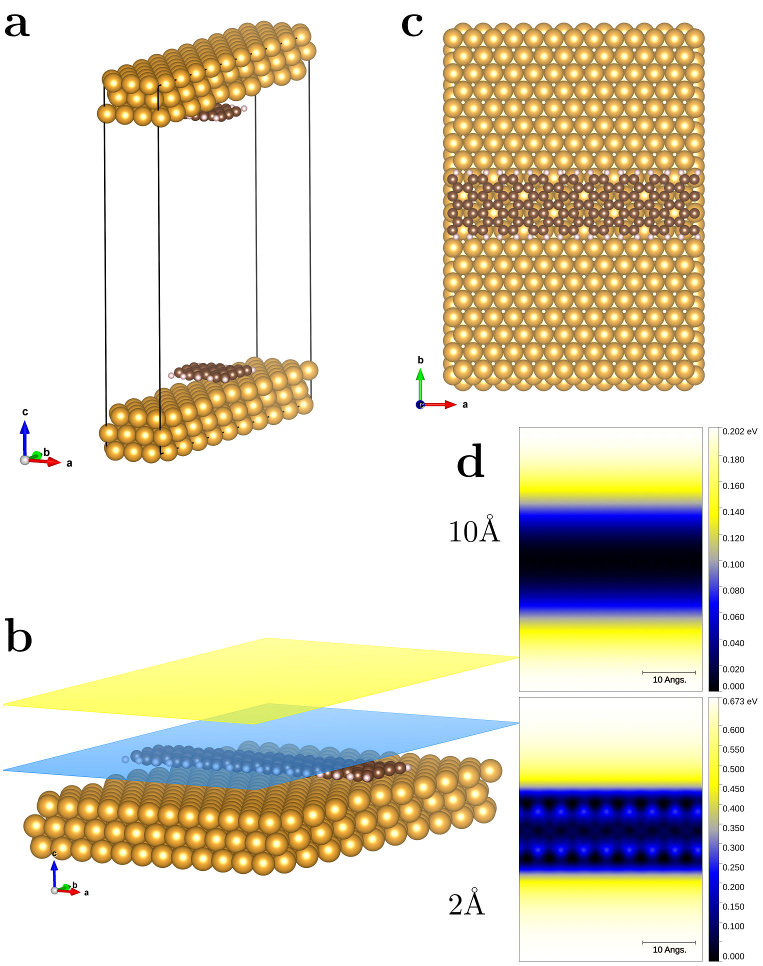

Figure S1: (a) Orthorombic unit cell used in the calculations. (b) The horizontal planes used to determine the LCPD maps for $s = 2$Å (blue) and $s = 10$Å (yellow) are indicated above a 4×1 surface cell. (c) Top view on the same cell and (d) LCPD maps for the same cell and different values of s.

Force–distance data

Figure S2 shows force-versus-distance data derived from a frequency shift-versus-distance measurement. The frequency shift has been converted to force using Baratoffs method briefly described in [1]. The frequency shift-versus-distance data has been used to convert Δf data to distance data.

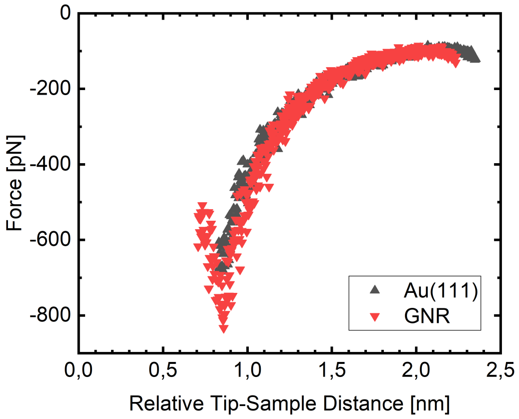

Figure S2: Force as a function of distance measured with a PtIr tip, f_0 = 247.00 kHz, c_L = 40 N/m, A = 2 nm, Q = 13000, V_{bias} = 144.5 mV, T = 120 K.

Calculated charge differences

The following electron densities have been generated:

- $\rho_{\text{GNR+Au}}$: electron density of the whole system,
- ρ_{GNR}: electron density of the isolated GNR with the same atomic positions as in the whole system, and
- ρ_{Au}: electron density of the isolated Au surface with the same atomic positions as in the whole system.

We compute

$$\rho_{\text{diff}} = \rho_{\text{GNR+Au}} - (\rho_{\text{GNR}} + \rho_{\text{Au}})$$

in order to visualize the charge transfer between the GNR and the surface, similar to the approach in [2]. The resulting ρ_{diff} is shown in Figure S3. As one can see, the largest differences are close to the GNR, but there are also some in the first and second Au layers.

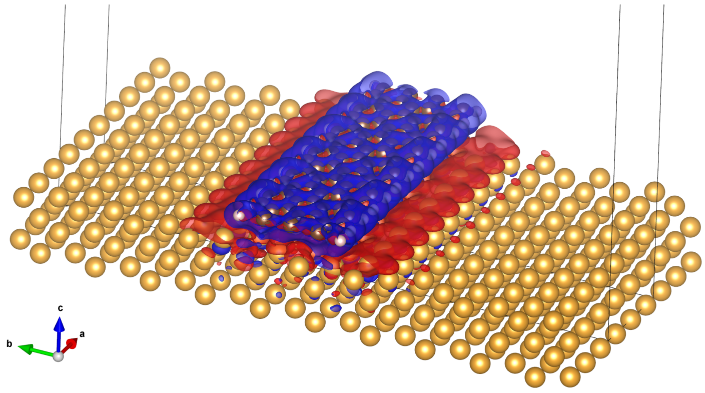

Figure S3: Isosurfaces of ρ_{diff} (details see text). The blue surface corresponds to -10^{-4} eÅ^{-3} the red to $+10^{-4}$ eÅ^{-3}.

References

1. Pfeiffer, O.; Bennewitz, R.; Baratoff, A.; Meyer, E.; Grütter, P. *Phys. Rev. B* **2002**, *65*, 161403. doi:10.1103/PhysRevB.65.161403

2. Liang, L.; Meunier, V. *Phys. Rev. B* **2012**, *86*, 195404. doi:10.1103/PhysRevB.86.195404
